# Systematic co-development and testing of a digital behaviour change intervention for osteoarthritis and physical activity: Theoretical mapping and acceptability study

**DOI:** 10.1177/20552076231204425

**Published:** 2023-10-06

**Authors:** Alice Berry, Candy S McCabe, Sarah Muir, Nicola Walsh

**Affiliations:** 1 Centre for Health and Clinical Research, University of the West of England, Bristol, UK; 2 Dorothy House Hospice, Bradford-on-Avon, Wilts, UK; 3 MRC Lifecourse Epidemiology Centre, 7423 University of Southampton, Southampton, Hampshire, UK

**Keywords:** Digital behaviour change intervention, arthritis, physical activity, intervention mapping, intervention development, usability

## Abstract

**Objective:**

Osteoarthritis (OA) affects 8.75 million people in the UK. Physical activity (PA) is recommended as a core treatment, yet nearly half of people with OA are inactive. Accessible and user-friendly interventions are needed to motivate people with OA to be active. Digital behaviour change interventions (DBCIs) might help to support people with OA to self-manage their own levels of PA. The aim of this project was to co-develop and test a DBCI to motivate people with OA to be active.

**Methods:**

A mixed methods design was adopted to build the theoretical foundations, develop, and test a complex DBCI. Two patient research partners with lived experience of OA were recruited onto the project team to assist with intervention development, which was guided by the intervention mapping (IM) approach. Interviews and think-aloud sessions were then used to explore attitudes, values, and perceived effectiveness of the website.

**Results:**

The IM approach enabled the development of a prototype website to be illustrated in a clear and transparent way, showing a link between the practical materials adopted within the website and the theoretical constructs they were attempting to change. Potential users highlighted the importance of clear, easy-to-understand information, focusing on enjoyment and social connectedness.

**Conclusions:**

DBCI development should be based on theory, adequately described, and thoroughly tested with potential users to understand how they might choose to integrate digital interventions into everyday life.

## Introduction

Around 8.75 million people in the UK are impacted by osteoarthritis (OA),^1^ a musculoskeletal condition primarily affecting the knees and hips, causing joint pain, muscle weakness, depression, anxiety, and low confidence.^2,3^ Supporting people with OA to self-manage their condition can improve knowledge and performance of self-management behaviours and increase levels of self-efficacy (confidence in one's ability to carry out a behaviour).^4^ Treatment guidelines encourage individualised self-management strategies and place emphasis on the importance of incorporating exercise and physical activity (PA).^5^

The use of digital technology to deliver self-management interventions is increasing, yet it is often unclear how such interventions have been developed and how content aligns to theory. The COVID-19 pandemic demonstrated the value of digital health interventions whilst also highlighting digital divides, with 9 million people still requiring help to use the internet.^6^ Given the unprecedented switch to digital methods to deliver healthcare, the issue of transparency, acceptability, and understanding how content within digital interventions is underpinned by theory becomes even more important.

Research is needed to demonstrate how individual components of interventions work and are used by participants.^7^ Furthermore, developing digital behaviour change interventions (DBCIs) with a person-centred and iterative co-design approach to progressively refine the DBCI are needed, to improve acceptability and usability,^8^ and ensure transparency. The aim of this project was to develop and test the acceptability and usability of a DBCI to motivate people with OA to become and stay active and illustrate this process using intervention mapping (IM).

## Methods

A sequential explanatory mixed methods research design was adopted to build the theoretical foundations, develop, and test a complex DBCI. A pragmatic epistemological position was adopted throughout the project, where the concern was with identifying what works, and focusing on solutions, using a range of methods to learn about, and understand the problem.^9^ In this instance, quantitative data provided a general understanding of the problem, and qualitative data began to refine and explain the statistical results. Each set of data was considered to have an equally important role and was therefore given equal weighting. Two patient research partners (PRPs) with lived experience of OA were recruited onto the project team to assist with intervention development, which adopted the IM approach to illustrate the process. Methods included incorporating the findings of a systematic review,^10^ and quantitative data from a survey,^11^ to guide the co-design and co-development stage (illustrated using IM) and a testing stage, which involved ‘think-aloud’ sessions and interviews to understand acceptability and usability at this early development stage. Table 1 provides an overview of the research methods utilised within each step of the development process.

**Table 1.** Steps of the intervention mapping approach.

Intervention mapping stages	Description	Research methods
Step 1: logic model of the problem	Conduct needs assessment Create logic model of the problem Describe context of intervention State programme goals	Systematic literature review Survey Secondary data analysis
Step 2: programme objectives, logic model of change	State programme aim and objectives Select identifiable determinants of behaviour Construct matrices of change Create logic model of change	Mapping exercise Team consensus exercise
Step 3: programme design	Generate programme themes, components, scope, and sequence Identify and select relevant behaviour change techniques Select practical applications to deliver chosen techniques	Team consensus meetings Mapping exercise Development of design document
Step 4: programme production and early testing	Refine programme structure Plan programme materials Draft messages, materials, protocols Pre-test, refine, produce materials	Digital prototype development Usability testing (think-aloud sessions) Acceptability testing (in-depth interviews)

### Intervention mapping

IM provides a framework to illustrate the move from theory and evidence into design and practice,^12^ showing the link between practical materials adopted within a digital intervention and the theoretical constructs it attempts to change. IM aligns well with the MRC framework for developing complex interventions^13^ and has been used to develop interventions in similar settings.^14–16^ The first four steps of IM were systematically applied in this project.

### Ethical approval

Ethical approval from the Faculty Research Ethics Committee at the University of the West of England was granted in two stages: August 2015 (UWE REF: HAS/15/06/184) for the survey study (step 1) and May 2018 (UWE REF: HAS/18/04/140) for usability and acceptability testing (step 4).

### Step 1: Logic model of the problem

Step 1 involved understanding and describing the problem that the intervention focuses on, by conducting a needs assessment to understand the context of the intervention, creating a logic model of the problem, and stating the overarching programme (or intervention) goals. This step used data from separately published studies ((a) findings of a systematic literature review, which aimed to determine the effectiveness of digital interventions for promoting PA in people with OA^10^ and (b) quantitative data from a cross-sectional survey that explored the role that beliefs, motives, and gains have on PA participation^11^). These earlier stages of the project have been published previously so are not repeated here. This data was combined with broader scoping of the literature (including a review of most recent clinical guidelines for self-management of OA, PA guidelines, broad determinants of PA within OA population, and review of key psychological theories of behaviour change) to provide insight into the problem of physical inactivity in this population, from a motivational perspective.

### Step 2: Programme objectives, logic model of change

This step involved developing a foundation for the intervention by specifying who, what, and how change would occur as a result of the intervention.^17^ This included stating the programme aim and objectives, selecting identifiable determinants of behaviour, constructing matrices of change, and creating a logic model of change. Methods adopted during this step included the following: (a) a comprehensive mapping exercise where components of effective interventions identified in the systematic literature review (step 1) were mapped to the Behaviour Change Technique Taxonomy (V1) (BCTTV1),^18^ which highlighted the main areas of focus and guided the formation of the overall aims of the intervention, and (b) a team consensus exercise was held to develop change objectives (COs). COs represent the pathways for the most immediate changes in the identified behaviour,^17^ in this instance identifying what guidance and advice were needed to support the relevant behaviour change (PA engagement).

### Step 3: Programme design

The process of programme design was steered by a number of team consensus and mapping exercises, which guided the development of a design document to conceptualise the intervention. The team worked through the aims and objectives of the intervention, directed by clinical guidelines, and discussed what content should be included and how it might be presented. Several guiding principles and key themes were created, and the scope and sequence of the intervention were defined. Potential practical applications were shown to the team, and choice of applications discussed. A mapping exercise was carried out where behaviour change techniques (BCTs) were linked to the COs created in step 2. Ideas for pages, headings, and practical materials discussed in the team consensus meetings were collated into the design document.

### Step 4: Programme production and testing

This step included developing the prototype intervention, as well as formal testing with potential users, using qualitative methods (including the think-aloud approach and in-depth semi-structured interviews) to explore usability and acceptability.

#### Step 4a: Prototype development

The purpose of this step was to produce creative messages and materials for use in the prototype intervention (website). The challenge was to successfully translate the BCTs and practical applications detailed in the design document (step 3) into creative, operational materials that promoted and supported the key messages of the intervention. This step involved three sub-tasks: (a) paper prototyping, to explore if available materials matched the COs and BCTs and to provide an early representation of how a material might be displayed; (b) digital prototype creation, to review materials in an early semi-functional prototype so that team members could comment on suitability and appearance; and (c) testing with PRPs, to explore general usability and functionality and to pilot the ‘think-aloud’ method prior to its use in step 4b (formal acceptability and usability testing).

#### Step 4b: Acceptability and usability testing

This step involved using qualitative methods to explore what a group of potential users thought about the digital health intervention, not only in terms of how easy it was to use but also, importantly, how acceptable the content was, including its relevance, coherence, perceived amount of effort required to use the intervention, and how it might be used as part of everyday life. Two qualitative data collection methods were used: usability was explored using a ‘think-aloud’ method,^19^ and semi-structured interviews were carried out to investigate how acceptable the content was to people with OA.

##### Usability: ‘Think-aloud’ sessions

The think-aloud protocol is a widely used method for evaluating the usability of digital interventions.^20^ The method investigates what will happen when real users start to use an intervention, by observing them using the intervention in real time.^19^ During recorded sessions, users ‘interact’ with the intervention while verbalising their thoughts, feelings, and actions, providing detailed insight into potential problems encountered by users, which can be used to inform future iterations and improve usability.^20^ The lead researcher (AB) completed the think-aloud sessions. Prior to the session, each participant was given a short introduction to the intervention. Once the session began, the participant was encouraged to talk out loud, describing their thinking whilst navigating the intervention or completing tasks. Notes were taken, and sessions were audio/video recorded to observe how the participants interacted with each of the web pages, and so that any user–computer interaction problems could be identified.

##### Acceptability: Semi-structured interviews

Each think-aloud session was combined with a semi-structured interview to explore how acceptable the intervention was to potential users. The interview schedule was developed in accordance with the validated^21^ theoretical framework of acceptability (TFA),^22^ which defines acceptability as a ‘multi-faceted construct that reflects the extent to which people delivering or receiving a healthcare intervention consider it to be appropriate, based on anticipated or experiential cognitive and emotional responses to the intervention’, and consists of seven constructs: affective attitude (how an individual feels about an intervention), burden (the perceived amount of effort that is required to participate in the intervention), perceived effectiveness (the extent to which the intervention is perceived to be likely to achieve its purpose), ethicality (the extent to which the intervention has good fit with an individual's value system), intervention coherence (the extent to which the participant understands the intervention and how it works), opportunity costs (the extent to which benefits, profits, or values must be given up to engage in the intervention), and self-efficacy (the participant's confidence that they can perform the behaviour(s) required to participate in the intervention).^22^

##### Recruitment and data analysis

An invitation to participate was extended to 80 participants who took part in the study in step 1 (and had consented to be contacted about future aspects of the project). Potential participants replied to the initial email within 2 weeks if interested; no follow-up reminders were sent. Inclusion criteria included a self-reported diagnosis of OA and access to the internet. The lead researcher (AB) carried out a home visit, and written informed consent was collected in advance of data collection. Think-aloud sessions and interviews were audio recorded, and all data were stored on firewall-protected, encrypted UWE computers and anonymised using unique ID numbers. Data from the think-aloud sessions were used to identify and review user–computer interaction problems. Recorded data from the interviews were transcribed and analysed using deductive thematic analysis.^23^ Data were coded deductively, TFA^24^ guided by the six steps of thematic analysis.^25^ The concept of data saturation is not consistent with the values and assumptions of thematic analysis adopted within this study, so there was no intention for data saturation to be achieved. Three team members coded one interview independently to check for consistency. In cases where multiple codes were present, AB made the final decision about the dominant code(s). Themes were then identified across all data.

## Results

### Step 1: Logic model of the problem

Data from the systematic literature review and survey were combined to provide an overview of the problem of physical inactivity within the OA population, from a motivational perspective. Key findings highlighted the importance of both self-efficacy and autonomous motivation on PA engagement in this population. These data were used to develop a logic model of the problem (see Appendix 1) and overarching programme goal of the intervention which was the following: *To provide tools (via a digital platform) which promote both autonomous forms of motivation and increase self-efficacy for PA, to facilitate sustained engagement in PA for people with OA.* Elements of both theoretical concepts^26,27^ were adopted as the determinants of behaviour.

### Step 2: Programme objectives, logic model of change

The mapping exercise linked components of the effective interventions identified in the systematic literature review^10^ to the BCTTv1^18^ highlighting the main areas of focus and helped to guide the formation of the aims of the intervention (see Appendix 2). Table 2 shows the aims and objectives of the intervention. Information from the needs assessment was used to guide the formulation of related performance objectives (POs) (i.e. what the participant needs to do to perform the health-related behaviour) for each aim.

**Table 2.** Aims and objectives of the intervention.

*Knowledge/skills*: To provide the user with sufficient knowledge about the benefits of PA for OA and access to appropriate resources, enabling the user to develop sufficient skills to carry out their chosen PA.	PO 1.1: Understand and accept the benefits of PA for OA. PO 1.2: Understand how to select and safely perform their chosen type of activity. PO 1.3: Understand that normal physiological responses (such as pain) can be experienced and how to respond.
*Action planning*: To provide the user with the appropriate tools to formulate and self-monitor specific, measurable, achievable, realistic, timely (SMART) goals, including the ability to review and update them when necessary.	PO 2.1: Learn about and set SMART goals for PA, using pacing/graded tasks. PO 2.2: Learn about self-monitoring and updating goals. PO 2.3: Acknowledgement of past successes of PA. PO 2.4: Problem solving and planning for challenging times, including recognition of how others have overcome barriers.
*Support*: To enable the user to identify and develop supportive social links, providing a sustained supportive environment for maintaining PA.	PO 3.1: Accept the emotional and practical benefits of a social network of support (and plan for). PO 3.2: Identify new social network links – friends/family/active others with OA. PO 3.3: Accept that one's own behaviour can be an example for others to help them to be physically active.

During the team consensus meeting, team members were asked to consider which determinants they felt needed to be changed for each programme objective to be met.

**Figure 1.**
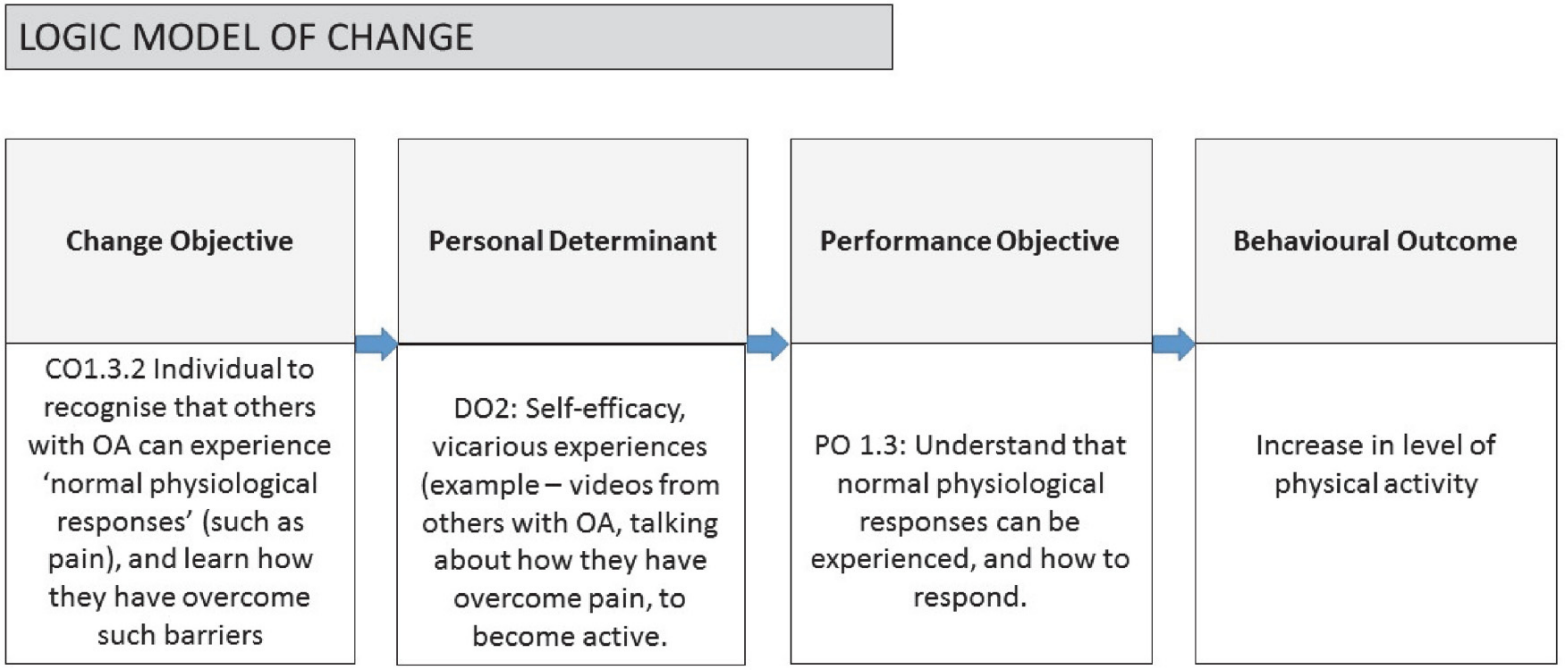
Logic model of change. CO: change objective; DO: personal determinant; PO: performance objective.

All members voted on each potential CO, votes were collated, and those with a majority were identified. The lead researcher (AB) wrote the COs following a review of guidance and published literature relevant to self-efficacy, self-determination theory (autonomous motivation), and literature reporting similar interventions. A logic model of change was created (Figure 1) to illustrate how an individual CO is hypothesised to influence a personal determinant (DO) and how that is linked to a PO and resulting behavioural objective (Appendix 3 provides full details of all COs linked to POs).

### Step 3: Programme design

Team consensus meetings (majority agreement, as judged by the lead researcher, AB) provided an opportunity for the team to discuss ideas for possible intervention content. Key points about the main sections of the website were discussed, consensus was reached on the inclusion of practical content, and issues considered important to further explore during prototype testing were also described, for example, exploring optimal methods for developing social support and appropriateness of language such as ‘goal setting’. Guiding principles were developed, which included ensuring the website was simple, accurate, easy to navigate, familiar, focused on enjoyment, and included stories from others. BCTs were linked to COs created in step 2, and details of practical materials were added into a design document (extract in Appendix 4).

### Step 4: Programme production and early testing

#### Step 4a: Prototype development

A series of paper prototypes were created to provide an early representation of how material might be displayed on website pages. The first version of the prototype website was then created using the online software package AXURE XP (Version 8). A consensus meeting with AB and PRPs took place to discuss the appropriateness of visual presentation of content. Several changes were made to the content and layout, including removing text to make sentences clearer; pages were simplified and consolidated to reduce the overall number of pages; the order of pages was updated to improve website flow. Separate meetings were also held with each PRP to pilot the think-aloud method and to allow for general comments about usability and functionality. Additional small changes were made, for example, pages were simplified, and key messages were made clearer. A final version of the prototype was then created (screenshots included in Appendix 5).

#### Step 4b: Acceptability and usability testing

Think-aloud sessions and in-depth interviews were carried out with seven participants, which is considered a sufficient sample to gain a thorough understanding of key issues with an intervention.^28^ Eighty-six per cent (*n* = 6) of the sample were female, and mean age was 73 years (SD = 13.2). Sample demographics are described in Table 3.

**Table 3.** Demographics of sample.

		*N* = 7
Gender	Female	6 (86%)
	Male	1 (14%)
Age (years)	Mean (SD)	73 (13.2)
	Minimum	60
	Maximum	93
Marital status	Married/partner	3
	Divorced/separated	1
	Widowed	3
Highest level of education	GCSEs or equivalent	1
	University degree or equiv.	1
	Post-graduate qualification	3
	None	2
Co-morbidities	Hypertension	2
	Mental health condition	1
	Other	1 (anxiety attacks)
Duration of OA (years)	Mean (SD)	10 (4.9)
	Minimum	3.5
	Maximum	20
Level of PA	Active (1–7 days)	6
	Inactive (0 days)	1
Days active per week	0	1
	1–4	0
	5–7	6
Minutes per day	Mean	115
	Minimum	20
	Maximum	300

##### Usability: ‘Think-aloud’ sessions

Think-aloud sessions ranged from 27 to 55 min. The primary focus was to explore what potential users thought about the design and functionality of the website. Three components of usability^29^ were explored: Learnability – could users accomplish basic tasks the first time they encountered the website? Errors – how many errors did users make, and could they easily recover? Satisfaction – How pleasant did participants find the design? Sessions were audio recorded, and notes were made throughout to describe how users chose to complete tasks and navigate the website.

Most participants learnt how to navigate the website quickly, though some did not complete the task boxes or click on buttons, which showed additional information as it was not clear to them that these buttons were interactive. Participants found the infographics useful and complimented the images used. Others suggested that animations would have been useful. A mock-up of a local map showing PA opportunities was praised, and participants described being keen to find out about local opportunities and to potentially ‘buddy-up’ with others local to them. Some participants questioned if the website would really capture future user's attention, suggesting that it could be more colourful, with less (textual) information, and more intuitive.

##### Acceptability: Semi-structured interviews

Five key themes were identified and are described in more detail below. Pseudonyms are used to describe participants.

###### Theme 1: Knowledge was valued, and beliefs about the benefits of PA for OA were positive

This theme illustrates the attitudes and perceived effectiveness of PA for potential users. Table 4 provides an overview of each of the sub-themes for theme 1. Some participants felt that the knowledge section of the website would most benefit those with a recent diagnosis. Attitudes towards PA were positive, and participants understood the benefits of PA for OA and placed value on it. Participants acknowledged the gap between intention and behaviour and described how they were more likely to carry on with an activity if they enjoyed it. Participants agreed with the importance of pacing; however, a number of them described their difficulties in getting it right.

**Table 4.** Sub-themes for theme 1 (knowledge was valued, and beliefs about the benefits of PA for OA were positive).

Sub-theme name and description	Example quotes	TFA construct(s)
*Depth of knowledge about OA*: We already have knowledge.	‘The average person doesn’t need to know all this stuff, there is only some of us that want to know what's going on here’. Anne, 63	Affective attitude
*Beliefs about the benefits of PA for OA*: We value the benefits of PA, but it becomes less of a priority when we are well.	‘I mean, I know what I should be doing, walking, and swimming, you know’. Betty, 85 ‘Every now and again the doctor will print out something, and I will do it, but once the issue has gone, you stop doing it… but you stop doing it, the issue reappears, and you don’t think to do that, do you’. Susan, 60	Affective attitude/ethicality/value
*Enjoyment is important*: We choose activities that we enjoy.	‘I did get myself a bike for myself in the house, had it, got rid of it, I didn’t enjoy it! I think that's probably why the walking is still going, because that is something that I do enjoy’. Susan, 60	Ethicality/value
*The difficulty of pacing and reviewing potential barriers*: Pacing is important and difficult to get right. We don’t think you should highlight the negatives (stumbling blocks).	‘Yep, that's the difficulty – with getting the right level of exercise, we’ve all been there, and done too much occasionally. I agree that little and often is often the best approach. That's been learnt through experience, and making mistakes’. Anne, 63	Ethicality/value Perceived effectiveness

###### Theme 2: The value (and burden) of setting goals

Table 5 provides an overview of the sub-themes in theme 2. This theme uncovered attitudes towards being active and also factors impacting levels of self-efficacy. The website prompted users to develop personalised specific, measurable, achievable, realistic, timely (‘SMART’) goals for PA. Some participants thought this section was too detailed, describing less structured, more abstract goals, often preferring to keep them in their heads rather than writing them down. Some participants were more positive about completing the goal setting exercise, though were unsure of the likelihood of returning to review them. For some, reflecting on past successes revived a sense of achievement and appeared to motivate them to re-start an old activity. Others felt that previous lifestyles were no longer achievable or realistic.

**Table 5.** Sub-themes for theme 2 (the value (and burden) of setting goals).

Sub-theme name and description	Example quotes	TFA construct(s)
*Attitudes and autonomy towards goal setting*: SMART goals are too much work.	‘We’re retired now, we don’t want to do goals anymore [laughs]! It's too work-like…. It's in my head or I have got my Google calendar which is my plan anyway, what am I doing this week and you know…. So, I already have a vehicle for my plan’. Anne, 63 ‘It's very childish isn’t it?’ Winnie, 93	Affective attitude
*Fear of failure/self-efficacy*: If you write down your goals, it's worse if you fail.	‘Probably that I don’t want to write it down because when I don’t achieve it, I don’t like failure’. Susan, 60	Self-efficacy
*Age and past successes*: Relevance of past successes varied for different ages. Contrasting results. We’re older now, we have a different pace of life.	‘Oh, I like this, I like this idea (recording past successes), this has got me straight away. But then I suppose others might say that's not obtainable. I would look at that and think ‘yes!’ I’ve got to do Scafell, Ben Nevis, well, I did Snowdon…’. Patricia, 64	Affective attitude/self-efficacy

###### Theme 3: The impact of competing life priorities

This theme was important in understanding the external pressures that participants felt and provides some explanation of the competing opportunity costs that appeared to affect whether the participants felt able to engage with the website. Table 6 provides an overview of the sub-themes.

**Table 6.** Sub-themes for theme 3 (the impact of competing life priorities).

Sub-theme name and description	Example quotes	TFA construct(s)
*Our lives are full*: Family, children, grandchildren, elderly parents, work. PA/exercise is not a priority.	‘I don’t think about exercise, I think about what I like to do, because that's why I’m alive, to enjoy myself…. I try to build in my exercise into my everyday so that it doesn’t become a chore, although we do it depending on what other activities are going on, erm… being a pretty busy family’. Anne, 63	Opportunity costs
*Not enough time, and the impact of interruptions*: Life is busy and unpredictable, routine is difficult.	‘Committing to getting to a particular class at a particular time is where I would find difficulty…. its Sod's law says that's it's on a Tuesday. Tomorrow I’m not going because I’ve got a XXXX, normally, we’ve met on a Wednesday… things happen…’. Sylvia, 65	Opportunity costs
*Access to local services*: I can/cannot get to local classes and groups.	‘Well we haven’t got a local shop so I couldn’t walk to that… I have done Pilates and I used to go to a group but I don’t know whether there is a group that I can go to’. Winnie, 93	Burden/intended use

###### Theme 4: Being active with others and social support

A social support section of the website included ideas for developing practical support to help maintain PA. Participants were keen to explore options for linking up with others ‘who are like me’ but were less positive about using online forums to connect with others. Table 7 below provides an overview of the sub-themes.

**Table 7.** Sub-themes for theme 4 (being active with others and social support).

Sub-theme name and description	Example quotes	TFA construct(s)
*The enjoyment of being active with others*: I don’t like being alone.	‘So many people think they can do it on their own, but I realised, I’ve got a Pilates machine down there, it’s been sitting there for 4 years, hasn’t been used, but I’ll go to the Pilates class. You almost need a neighbour… or the person across the road’. Patricia, 64 ‘It’s so much nicer when you’re doing it with somebody else…’. Susan, 60	Affective attitude. Ethicality/value
*Relatedness*: They must be like me.	‘… like 6 weeks at the hospital (exercise group), once that was over, that was it. Although we did all start having a cup of tea afterwards in the cafe, but it didn’t continue. I mean I was out of their age group to be honest with you… I was one of the youngest ones there’. Susan, 60	Affective attitude.
*No to Facebook!* We’re keen to link up with people face-to-face.	‘Oh, I hate social networks… laughs, it doesn’t appeal to me at all, no’. Bernard, 81 ‘I think I am happier seeking it out myself. I am a bit mistrustful of a lot of these group things on computers’. Sylvia, 65 ‘What I need to do is find out if there are a group of people doing things, where they can then say, oh, come and join us… that’s what I need’. Betty, 85	Affective attitude. Ethicality/value
*The impact of personal relationships*: We exercise together/I have no support, it's hard	‘I am so lucky that I have a fantastic husband…. we do tend to talk about what we are going to do during the day and both of us are trying to build in exercise with everything, and if we have a day that we can’t exercise then we make up for it by doing a long walk or something’. Anne, 63	Affective attitude
*Self-efficacy for social interaction*: I don’t want/need this type of support and haven’t got much to offer to others.	‘So perhaps if, like, local groups, I say local groups, we do have a local walking group, but I’d never have the courage to go, yeah, I don’t know why, I just don’t’. Susan, 60	Self-efficacy. Perceived effectiveness.

###### Theme 5: Maintaining professional support whilst independently self-monitoring

Participants placed value on professional support; however, they did not want to be disturbed with too many notifications and updates. Table 8 provides an overview of the sub-themes.

**Table 8.** Sub-themes for theme 5 (maintaining professional support whilst independently self-monitoring).

Sub-theme name and description	Example quotes	TFA construct(s)
*Value of professional support*: We would like to maintain a link to professional support	‘My preference is actually just to talk it through with someone, is there anything else I should be doing really. It's that because I can think about what I need to do and my motivation comes from within but as I said recently I got into a pickle and couldn’t understand why I couldn’t get out of pain and I realised, when I spoke to somebody that I hadn’t done the pacing properly’. Anne, 63	Ethicality/value.
*Autonomy to self-monitor*: We want to be in control of how often we receive a notification/email/text.	‘No, I don’t want you to text me, because I review myself’. Sylvia, 65 ‘It would be nice to have the choice. Because I did switch off the notifications because I am not keen, you get too many. I’ve got a Garmin activity watch that used to drive me nuts, because I be sitting watching a film, and it would tell me to move, so that's no good’. Patricia, 64	Affective attitude.
*Self-efficacy for self-monitoring*: We don’t need help to monitor our goals, we have established ways of doing this.	‘I have tried the App on the phone, where you walk and it tells you how far you’ve walked, and all that. I did try that, but… the goals are a little bit high, that’s what I found, and like I said, I live on a hill, so wherever I go…’. Susan, 60	Perceived effectiveness. Burden/intended use.

**Figure 2.**
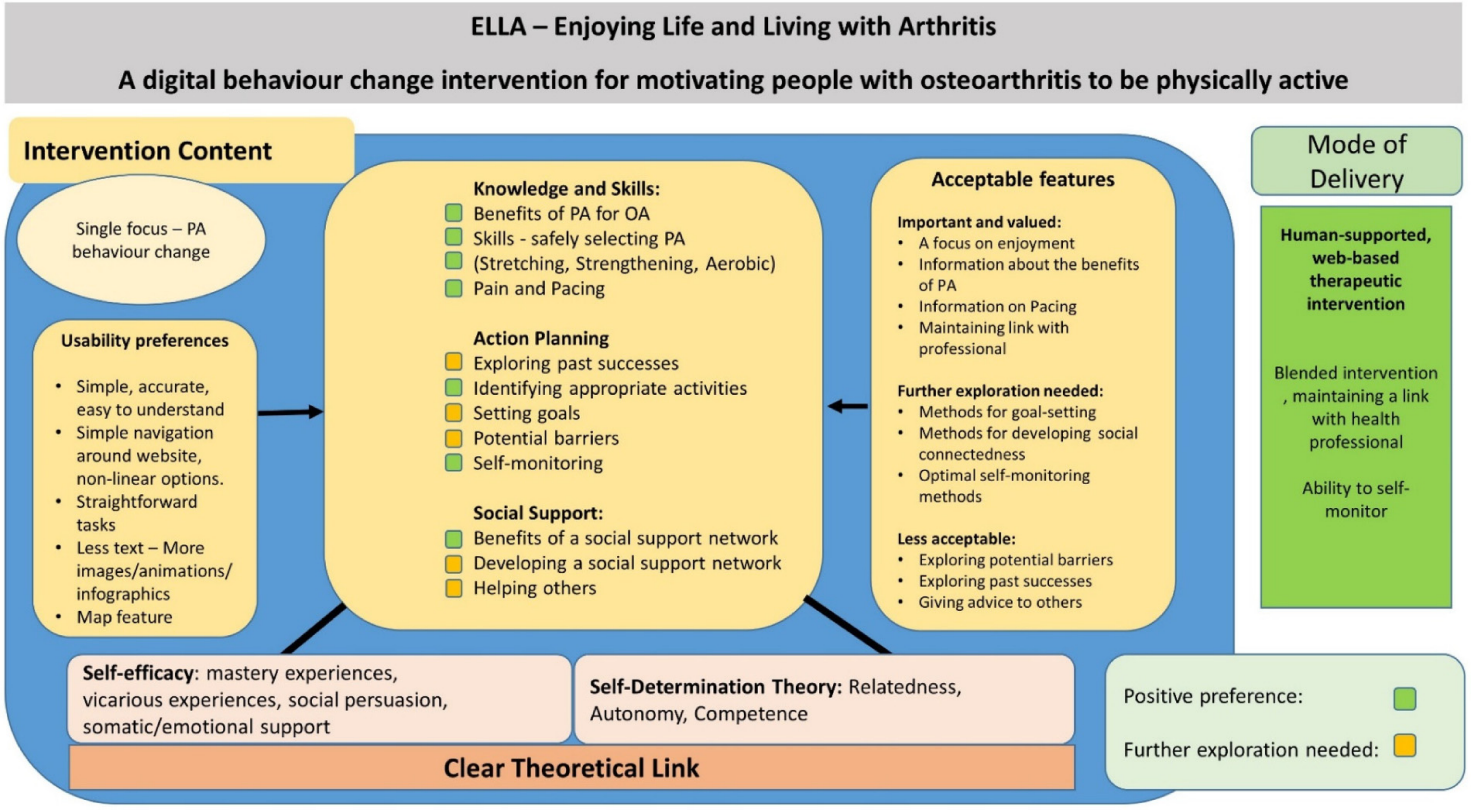
Model of the digital behaviour change intervention (DBCI).

Figure 2 illustrates a current model of the DBCI informed by this paper, illustrating how content was linked back to theoretical constructs. Incorporating the findings from all four steps of IM, it also highlights elements of the content reported to be acceptable and areas where future research is required.

## Discussion

This paper describes the development of a DBCI using the IM approach to illustrate how practical content links to theoretical determinants, emphasising a highly iterative process that integrates testing and consensus exercise within each stage. Refinements described throughout the process go some way to highlight which clusters of BCTs might be considered essential to include in PA interventions for this population. This paper also demonstrates the value of bringing together expert patients with first-hand experience of living with OA and clinical experts in the field of OA and pain management.

Novel use of the TFA to explore acceptability of the intervention emphasised the value of clear, easy-to-understand information, which focuses on activities that are enjoyable. It highlighted the importance of increasing social connectedness as well as the potential burden of goal setting and impact of competing life priorities, drawing attention to the potential impact that such issues have on engagement with digital interventions. Acceptability has become a key consideration in the design, evaluation, and implementation of healthcare interventions, yet without a shared understanding of what acceptability refers to it remains difficult for intervention developers to assess it effectively.^22^ The prompting of more intangible opinions and thoughts enabled a deeper understanding about the attitudes and values of the sample group. Some intervention techniques may be effective when tested in an RCT but not widely acceptable to the target audience, whilst other techniques might be highly acceptable but show smaller effect sizes.^30^ Better understanding of acceptability could produce more effective interventions, in turn improving adherence to the behaviour. Acceptability testing also highlighted the importance of social support and connectedness (relatedness, etc.). A key finding from potential users was the desire to link up with others ‘like me’. This area has perhaps been overlooked previously in digital interventions, where the focus has been on setting individual action plans and self-monitoring goals.

Future interventions that attempt to create an optimal environment for autonomous motivation and self-efficacy for PA in a similar population might consider the following: enjoyment – support the user to choose an activity that they think they will enjoy; social connectedness – integrate tools that allow the user to identify local groups/buddies to encourage activity with others and to develop relatedness to others; action planning – support the user to form a plan of action, being mindful of the time/effort required to develop the plan/goals; keep as simple as possible; and allow the user to create their own structure to strengthen autonomy or consider other goal-setting options as an alternative to SMART goals^31^; professional link/blended care – integrate options for linking up with professionals; recognise that competing life priorities (as well as OA-specific barriers) will affect engagement with PA; support users to plan for interruptions; and acknowledge that other people have highlighted the difficulties of competing life priorities when trying to make plans; and knowledge/skills/PA guidelines – ensure all information about PA is simple, accurate, easy to understand, and up to date. A key message to get across should be any movement is good; include information on pacing and graded activity; and incorporate advice about being active despite the presence of pain. We also recognise that the findings of this testing phase provided guidance for subsequent versions of the website created within this project.

### Wider determinants of PA in OA

It is acknowledged that despite SE and autonomous motivation contributing to the understanding of PA maintenance in OA, it is unlikely that these concepts alone provide a complete understanding of all of the factors impacting PA behaviour in this population.^17^ It is important to recognise that behaviour is affected by wider social and environmental influences.^32^ Future interventions might consider integrating other models of behaviour change, which include the wider determinants of PA behaviour (such as the Health Action Process Approach (HAPA) model,^33^ the Physical Activity Maintenance (PAM) model,^34^ or the Social Ecological model^35^) in an attempt to understand other aspects of behaviour that contribute to long-term engagement with PA. There is a need to create supportive environments in which people can operate, yet provide individuals with the psychological tools to change and regulate their own behaviour.^32^ This involves taking a step back and acknowledging the importance of wider determinants such as social systems and the physical environment, as well as considering how local policy might contribute to PA levels across different regions of the UK.

### Limitations

The participant samples recruited in steps 1 and 4 were predominantly active, highlighting the issue of potential recruitment bias. Like other research, this project highlights the challenges of recruiting inactive participants into PA research studies.^36,37^ It is recognised that people already meeting PA recommendations are often attracted to PA interventions, even though they are typically not the target audience for such interventions.^37^ Future research should ensure a more balanced sample and include an adequate representation of non-active participants. Reviews of existing PA interventions demonstrate that current recruitment strategies tend to engage predominantly white, middle-class, middle-aged women unless they are clearly designed to target specific characteristics, such as gender or ethnicity.^36,38^ There continues to be a paucity of PA studies that include participants from varying socio-economic or ethnic groups.^39^ Digital behaviour change studies need to ensure that they recruit representative samples that cover a wider range of education level and socio-economic status, alongside other demographics such as age, gender, and ethnicity, to avoid selection bias.^7^ Future research should focus on establishing the beliefs, attitudes, and needs of sub-groups who could benefit the most from PA interventions. Strategies for engaging and involving inactive participants from under-represented groups with PA research need to be further developed.

## Conclusions

Developing digital health interventions in a dynamic and evolving environment presents new challenges, particularly around engagement, both with the technology and the intended behaviour. Whilst the COVID-19 pandemic demonstrated the value of digital health interventions, it has also highlighted a growing inequality between population sub-groups. This highlights the importance of understanding the factors that affect the uptake, sustained engagement, and acceptability of digital interventions to ensure that this digital divide and broader inequalities are not widened.

There is significant potential for digital interventions to engage people with PA. The current landscape of digital health is calling out for more rigorously developed, evidence-based interventions, which meet the needs of specific populations and can provide useful, acceptable tools, which guide engagement with healthy behaviours such as PA. Future interventions should be underpinned by theoretical reasoning and described in relation to accepted behaviour change taxonomies.

This paper offers an example of how this can be done, providing a useful blueprint for future digital intervention developers. This depth of development should become commonplace in the future, making it easier for the ‘active ingredients’ of an intervention to be illustrated and optimised and facilitating a smooth progression to subsequent process and implementation evaluations.

## Appendix

**Appendix 2:** Development of programme aims.

BCTs identified in needs assessment (coded to the BCTT v1^18^)		BCT grouping	Guidance for programme outcome (AIMS)
1.1	Goal setting (behaviour)	Goals and planning	Action plan
1.2	Problem solving		
1.4	Action planning		
1.5	Review behaviour (goals(s)		
1.6	Discrepancy between current behaviour and goal		
1.9	Commitment		
2.3	Self-monitoring of behaviour	Monitoring	Action plan
2.4	Self-monitoring of outcome(s) of behaviour		
2.7	Feedback on outcomes of behaviour(s)		
3.2	Social support (practical)	Social support	Social support
3.3	Social support (emotional)		
4.1	Instruction on how to perform the behaviour	Knowledge	Knowledge/skills
4.2	Information about antecedents		
5.1	Information about health consequences	Natural consequences	Knowledge/skills
6.1	Demonstration of the behaviour	Comparison of behaviour	Knowledge/skills
7.1	Prompts/cue	Associations	Action plan
8.1	Behavioural practice/rehearsal	Repetition	Action plan
8.3	Habit formation		
8.7	Graded tasks		
9.1	Credible source	Comparison of outcomes	Knowledge/skills
10.4	Social reward (includes positive reinforcement)	Reward	Social support
11.2	Reduce negative emotions (includes ‘stress management’)	Regulation	Action plan
12.6	Body changes (strength training/relaxation)	Antecedents	Knowledge/skills
13.2	Framing/reframing	Identity	Knowledge/skills
13.3	Incompatible beliefs		

## Appendix

**Appendix 3:** Change objectives linked to performance objectives.

Performance objective	DO1: self-efficacy, mastery experiences	DO2: self-efficacy, vicarious experiences	DO3: self-efficacy, social persuasion	DO4: self-efficacy, somatic/emotional states	DO5: SDT, relatedness	DO6: SDT, autonomy	DO7: SDT, competence
**Knowledge/skills: to provide the user with sufficient knowledge about the benefits of PA for OA and access to appropriate resources, enabling the user to develop sufficient skills to carry out their chosen PA**							
PO 1.1: understand and accept the benefits of sustained engagement with PA for OA.	CO1.1.1: individual to have access to sufficient information so that they are able to understand how PA can help OA.	CO1.1.2: individual to understand how others (from a physiological perspective) have benefited from starting and continuing PA.	CO1.1.3: process – individual to receive persuasive prompts/advice.	Co1.1.4: process – messages to be focused on understanding of emotional states/mood.	CO1.1.5: individual to understand how others have benefited from continued PA	CO1.1.6: process – individual to have choice about what information they receive – i.e. more/less.	CO1.1.7: individual to have access to sufficient information so that they are able to understand how continued PA can help OA in the long term.
PO 1.2: understand how to select and safely perform their chosen type of activity.	CO1.2.1: individual to have access to sufficient information so they can select appropriate activity and carry it out safely.	CO1.2.2: examples of how others chose safe over unsafe activities.				CO1.2.6: process – have choice for more/less information.	CO1.2.7: individual to have access to sufficient information so they can select appropriate activity and carry it out safely.
PO 1.3: understand that normal physiological responses (e.g. pain) can be experienced and how to respond.	CO1.3.1: individual to reflect on previous/current experiences of physiological responses such as pain and consider options for how to respond.	CO1.3.2: individual to recognise that others with OA have experienced ‘normal physiological responses’ and how they have overcome barriers.		CO1.3.4: individual to recognise that others can experience ‘normal physiological responses’ and how they have overcome such barriers.			CO1.3.7: individual to understand what a ‘normal physiological response’ might feel like.
**Action plan: to provide the user with the appropriate tools to formulate and self-monitor SMART goals, including the ability to review and update them when necessary**							
PO 2.1: learn about, and set SMART goals for PA, using pacing/graded tasks.		C02.1.2: individual understands that others have benefited from setting SMART goals/and using pacing/graded tasks.	CO2.1.3: verbal encouragement about the benefits of setting goals.	CO2.1.4: individual understands the benefits of using pacing and graded tasks.	CO2.1.5: see CO2.1.2.	CO2.1.6: individual understand what a SMART goal is and to choose own SMART goals. And choice to decide how many goals etc.	CO2.1.7: individual confident to select appropriate goals/pacing/graded tasks for current level of PA.
PO 2.2: learn about self-monitoring and updating goals.	CO2.2.1: individual accepts the benefits of self-monitoring, and is confident to make a plan.	CO2.2.2: examples of others who have set up goals. How and when, have self-monitored their behaviour.	CO2.2.3: social encouragement from others, you can succeed at your goals – sharing of goals?	CO2.2.4: understand the emotional benefits of self-monitoring your goals – stories?	CO2.2.5: see CO2.2.2.	CO2.2.6: individual chooses how they monitor PA and how often they receive prompts.	CO2.2.7: see CO2.2.1.
PO 2.3: acknowledgement of past successes of PA.		CO2.3.2: individual to recognise the benefits of others who have explored their previous successes.	CO2.3.3: verbal encouragement about the benefits of exploring and recording past success.	CO2.3.4: individual to accept that exploring previous success can have a positive effect on emotional/somatic states.	CO2.3.5: see CO2.3.2.	CO2.3.6: process – individual to have a choice to record previous successes/or not.	CO2.3.7: individual to explore and recognise the value of previous personal successes.
PO 2.4: problem solving and planning for challenging times, including recognition of how others have overcome barriers.	CO2.4.1: individual to explore (and record) previous (and current) barriers and how they were (and might be) overcome.	CO2.4.2: accept that others have successfully overcome barriers and learn about how they have done this.	CO2.4.3: advice from others (verbal encouragement) about how others have successfully overcome barriers – tools and advice.	CO2.4.4: individual to understand how to overcome emotional and somatic responses/barriers to PA (mood, etc.).	CO2.4.5: relate to others who have successfully overcome barriers.		CO2.5.7: individual to understand and accept that barriers to PA can be overcome.
**Support: to enable the user to identify and develop supportive social links, providing a sustained supportive environment for maintaining PA**							
PO 3.1: accept the emotional and practical benefits of a social network of support (and plan for).	CO3.1.1: individual to plan for how social support might help with practical issues of PA		CO3.1.3: individual to accept the benefits that emotional and practical support	CO3.1.4: recognise the positive effects that a social network can have on emotional responses		CO3.1.6: process – individual to make own plan about how a social network might support them	CO3.1.7: individual to be accept the benefits of developing a social network
PO 3.2: identify new social network links – friends/family/active others with OA	CO3.2.1: individual to gain confidence identifying and contacting friends/family for support	CO3.2.2: individual to learn how others have benefited from support from family and friends	CO3.2.3: individual to accept the benefits of support from family and friends	CO3.2.4: individual to accept the emotional benefits of support from family and friends	CO3.2.5: individual to learn how others (like them) have identified and started new activities with others with OA	CO3.2.6: individual to identify/name those who can provide such support	CO3.2.7: individual to gain confidence in identifying and contacting friends and family for support
PO 3.3: accept that one's own behaviour can be an example for others to help them to be physically active	CO3.3.1: individual to identify positive personal outcomes that could help others with OA to be active	CO3.3.2: individual to learn about how shared experiences can help people with OA become active	CO3.3.3: encouragement from others, to share success stories	CO3.3.4: individual to accept the emotional benefits of helping others to be active.	CO3.3.5: recognise that one's own behaviour can help others to succeed.	CO3.3.6: process – option to share own personal experiences to help others	

## Appendix

**Appendix 4:** Extract of design document

Change objective codings	Page name	boldContent	BCTS (BCTTV1)
**Performance objective 1.1: understand and accept the benefits of PA for OA**			
CO1.1.1	What is OA?	*What is OA?* OA is a condition that affects the joints. 8.75 million People aged 45 and over in the UK have sought treatment for OA (ARUK, 2017). These can all make it difficult to use the joint normally, and do things like climbing stairs. (Two images from ARUK - normal joint, and joint with mild OA). *How does it affect my joints?* Surfaces of your joints become damaged so it doesn’t move smoothly. Cartilage covering the ends of the bones roughens and becomes thin. Bone at the edge of your joint grows outwards, forming bony spurs called osteophytes. The joint capsule may thicken and make extra fluid, causing your joint to swell. Ligaments (tough bands that hold the joint together) slowly thicken and contract as if they were trying to make your joint more stable. Two pictures from ARUK - One of normal joint, one with mild OA.	5.1 Information about health consequences.
CO1.1.1	Your symptoms can improve	*Your symptoms can improve*: Many people think OA is untreatable. This is not correct. There is no cure for the degenerative changes seen in the joints, but this does not mean there is nothing that can be done to help the problem. There is a lot you can do to reduce pain and maximise your ability to do what you want. Having a healthy lifestyle, which includes remaining active, and keeping your weight controlled, can make a significant difference. You can still lead a healthy, active life if you have arthritis. SYMPTOMS CAN INCLUDE: • Pain • Stiffness • Swelling • Grinding sensation when the joint moves	13.3 Incompatible beliefs. 12.6 Body changes
CO1.1.2	How can PA help my arthritis?	*Stories from others*: (VIDEO FROM healthtalkonline - myscrapbook - advice about PA from others. Story (as text) - FROM ARUK - MEL'S STORY (Source: ArthritisResearchUK (www.arthritisresearchuk.org). ‘My Symptoms Improved when I Became Active’ ‘I decided to exercise to keep mobile, to sleep better and stay positive. Shane, a personal trainer at the gym, knew about my arthritis and created a programme of weights, stretching and resistance training I could do without hurting my knees. I started slowly doing a little each time, stopping if anything hurt. Within weeks I was able to walk upstairs without pain’.	13.2 Framing/re-framing 6.2 Social comparison
CO1.1.3	How can PA help my arthritis?	*Keep moving* This approach can help you to feel in control, and to manage symptoms better in the long-term. It doesn’t matter how old you are, or how long it is since you last exercised. PA will help to maintain your joints and make you fitter and healthier.	15.1 Verbal persuasion about capability
CO1.1.4	How can PA help my arthritis?	If you have arthritis, regular PA has special benefits above and beyond the general benefits of improved health, including: Reduced joint pain and stiffness Improved joint circulation and decreased swelling Better balance, and greater comfort doing daily activities Stronger muscles to protect joints by improving stability and absorbing shock Higher energy levels, and improved mood.	5.1 Information about health consequences. 16.3 Vicarious consequences
**Performance objective 1.2: understand how to select and safely perform their chosen type of activity**			
CO1.2.1	What sort of PA should I do?	*What sort of PA should I do?* Try to find the right balance between rest and exercise. Little and often is usually the best approach. Stretching exercises can relieve stiffness and improve the range of movement in your joints. Strengthening exercises help to build stronger muscles, providing joints with greater stability, and help to improve balance. Aerobic (or cardiovascular) exercise is any exercise that increases your pulse rate and makes you a bit short of breath (for example, a brisk walk or swimming). This type of exercise can improve your general health and well-being, as well as reduce pain (aerobic exercise raises the levels of pain-relieving hormones called endorphins). INFOGRAPHIC - WHAT ARE THE GUIDELINES. HOW LONG, HOW OFTEN ETC.	4.1 Information on how to perform the behaviour. 4.2 Information about antecedent.
Co1.2.6	PROCESS	NEXT/BACK buttons, side menu etc.	
**Performance objective 1.3: understand that normal physiological responses can be experienced and how to respond**			
CO1.3.7	What if I experience pain?	You may experience discomfort when you are exercising, or for a day or two afterwards, especially if you are trying something new, or if it's something you haven’t done for a long time. Don’t worry, this does not mean you have harmed your joints, or that you should stop the activity. It is inevitable that with long-term joint conditions you will sometimes experience aches and pains during exercise or activity. What is important, is that you recognise the need to keep your joints moving, without overdoing it. You will gradually learn what level is right for you. DIRECTION - You will learn more about how to use PACING techniques, in the next section of this website.	5.1 Information about health consequences.

## Appendix 6: Study interview schedule

**Acceptability and Usability of a Digital Intervention to facilitate sustained engagement in physical activity for people with osteoarthritis**

**Semi-structured Interview – topic guide/interview schedule**

**(Attitude/feelings)** What are your thoughts about the website, now that you’ve had a go at using it?

**(Likes/Dislikes)** What did you like / dislike?

**(Attitude)** How do you feel about using it in the future?

**(Self-efficacy for int. use**) How confident are you that you can complete the TASKS on the website?

**(Presentation)** How did you feel about how the information was presented? I.e. the text, and videos - would you have liked it to be presented in a different way? E.g. animations?

**(Intervention Coherence)** Was it clear? Is there anything that could be clearer?

**(Ethicality/value) T**he aims of the website are to: increase your knowledge, help you to set goals, and to develop a support network - How important are these things to you, if at all? Is it relevant to you?

**(Perceived effectiveness)** How much do you think this website could help you to be active, or not?

What about over the longer term?

**(Burden/Intended use)** How do you feel about the amount of time and effort that was required to use the website?

How often do you think you might use the website in your day to day life? Over the long-term?

Would you use all elements of the website, or just some?

**(Opportunity Costs)** Do the potential benefits of using the website, outweigh the amount of time and effort needed to use it? Is it worth it?

**(Tailoring)** Would you have liked the information to be more personalised? Which bits?

**PROMPTS -** Stories/videos/exercise options

**(Self-Monitoring)** Do you think the website would help you to monitor your goals? – Or would you like additional tools to help you to monitor like – like linking up to your phone/pedometer/fitbit?

**(Professional support)** Would you like the website to include professional support? (face to face, or online)

**(Referral pathway)** How would you like to first access the website? GP/physio/directly online

**(Missing elements)** Is there anything missing? What else would you like?

**Closing**: Thank you very much for taking part, do you have any other reflections about anything we have discussed, or any other questions at all?
